# Development of Colorimetric and Ratiometric Fluorescence Membranes for Detection of Nitrate in the Presence of Aluminum-Containing Compounds

**DOI:** 10.3390/s18092883

**Published:** 2018-08-31

**Authors:** Hong Dinh Duong, Han Lae Kim, Jong Il Rhee

**Affiliations:** 1 School of Chemical Engineering and Research Center for Biophotonics, Chonnam National University, Yong-Bong Ro 77, Gwangju 61186, Korea; zink1735@gmail.com; 2 AquaTech Co., Ltd. 13 Yeongtong-ro 525 beon-gil, Yeongtong-Gu, Suwon 16706, Korea; tntp@hanmail.net

**Keywords:** oxazine–ethyl cellulose membrane, Devarda’s alloy, clay hydrotalcite, Nitrate detection, colorimetric and ratiometric fluorescence detection

## Abstract

In this study, a quantitative analysis of nitrate in aqueous solution was performed through the combination of an oxazine170 perchlorate–ethyl cellulose (O17-EC) membrane with aluminum-containing compounds. Aluminum of Devarda’s alloy (DA) or a clay hydrotalcite (HT) was employed for the reduction of nitrate to produce ammonia, and the produced ammonia was detected by the O17-EC membrane. The method of combining the O17-EC membrane with aluminum compounds has showed a broad detection range of nitrate. That is, the DA was combined with the O17-EC membrane and showed the linear nitrate detection ranges of 1–10 mM and 10–100 mM, while the O17-EC membrane immobilized with the clay HT showed a linear detection range of 0.1–1 mM nitrate. The visual color transition of the nitrate-sensing membranes at different nitrate concentrations was clearly observed under sunlight or irradiation of a light-emitting diode (LED) at an excitation wavelength of 470 nm (LED470).

## 1. Introduction

According to the Environment Protection Agency (EPA) of the United States (U.S.), nitrate (NO_3_^−^) is one of the parameters that must be determined for the evaluation of water quality in the environment, drinking water, and wastewater. Nitrate is toxic to humans, especially infants less than 6 months of age, at concentrations that exceed 10 milligrams nitrogen per liter (mg-N/L) in drinking water [1]. It also promotes the eutrophication of algae, leading to the pollution of lakes, rivers, etc.

As shown in the literature, a common method for nitrate detection is based on the reduction of nitrate to nitrite with copperized cadmium. The quantity of nitrite is then determined colorimetrically with Griess reagents [2,3,4,5]. The Griess assay has been accepted for many years and is still preferred today due to the continuous repairs and refinements that have been made to this method to achieve higher sensitivity [6,7] and a low limit of detection (LOD, e.g., LOD = 0.05 µM) [8]. Besides the Griess assay, the reductive reaction of nitrate is also exploited to develop electrochemical electrodes [9,10]. However, some problems cannot be overcome, such as poor sensitivity and irreversibility due to the cumulative electrode passivation effects [11]. The more sophisticated approach to the electrochemical detection of nitrate is the exploitation of biological catalysts. Nitrate reductase enzymes can significantly enhance both the sensitivity of the electrode and selectivity towards the reduction of nitrate [12,13]. However, the high cost and easily vulnerable sensing layers can limit their widespread application.

As another approach for nitrate detection, nitrate can be reduced to ammonia through reducing reagents, such as Devarda’s alloy [14,15] and zinc [16], and subsequently the produced ammonia is determined by typical methods [14,15,16]. In this approach, the reduction step of nitrate to ammonia is not complicated, but the detection method for ammonia is very important to obtain a wide linear detection range and a low limit of detection. Belcher et al. [15] use a technique called molecular emission cavity analysis (MECA) for ammonium nitrogen detection. This technique is rapid, precise, and free of interference, as it uses a hydrogen-based flame for generating molecular emission, and obtains a wide range of detection of nitrate, that is, 0.7–28.5 mM with an LOD of 70 µM. Su et al. [16] use a bulk acoustic wave impedance detector to detect ammonia from the reduction of nitrate and achieve a nitrate detection range of 0.0025–1 mM with an LOD of 1.7 µM. However, this approach has not attracted significant interest, probably due to the significant success of the Griess assay, the commercial availability of nitrate-ion-selective electrodes (ISEs), or the continuous development of modified versions of ISEs [17,18].

Despite the situation involving nitrate detection via ammonia products, in this study, we present a fluorescent membrane, specifically, the oxazine 170 perchlorate–ethyl cellulose (O17-EC) membrane for sensing nitrate in aqueous solution. Studies on fluorescent sensors for nitrate detection have not attracted wide interest, even though a few articles show positive results for nitrate detection. However, these results are far from reaching the standards of real applications. That is, Lee et al. [19] use a postcolumn fluorescence detector in HPLC to detect the fluorescence of Ce(III), which is created from the reaction of Ce(IV) with nitrite produced from the nitrate reduction. This method is very complicated but shows a detection range of 1–100 µM as well as an LOD of 5.2–15 pM. Chen et al. [20] synthesize a fluorescent probe that reacts with nitrate to turn on this probe’s fluorescence signal via a technique called aggregation induced emission (AIE). This probe shows a detection range of 0–0.1 mM with an LOD of 0.475 µM. However, the AIE technique is a limited method because of interference during operation. The toxicity of the probe is still not clearly understood, and the use of a probe to analyze samples in the environmental field is not preferred. The fluorescent sensors should be fabricated using sensing membranes, which can continuously measure and exhibit high reversibility for online monitoring. However, Kim et al. [21] developed a disposal optical film for nitrate detection with a detection range of 10^−5^–10^−1^ M, a high LOD of 10^−3^–10^−4^ M, and low sensitivity. Thus, anyone can understand the state of the art for a nitrate detection fluorescent sensor that is yet to be completed, is still missing from the literature, and needs quality improvement.

Herein, we exploited the reduction of nitrate to ammonia via reducing reagents containing aluminum, such as Devarda’s alloy or clay hydrotalcite. The produced ammonia was then determined using an ammonia-sensing membrane, i.e., the oxazine 170 perchlorate–ethyl cellulose (O17-EC) membrane. The O17-EC membrane was fabricated for ammonia detection as shown in our previous study [22], in which the O17-EC membrane was shown to be an excellent membrane for ratiometric measurement as well as to have high sensitivity to ammonia via a typical mechanism of the proton donation and reception between the weakly acidic oxazine and the weak base ammonium, respectively. The O17-EC membrane combined with different nitrate reduction steps created low and high detection ranges of nitrate. We proposed a simple and effective ratiometric fluorescence method for nitrate detection in terms of the easy fabrication of the sensor, fast measurement, high sensitivity, and high throughput of screening samples. Colorimetric measurements of the sensing membranes for the primary evaluation of nitrate in solution are performed as well.

## 2. Materials and Methods

### 2.1. Materials

Oxazine 170 perchlorate (O17), ethyl cellulose (EC), Devada’s alloy (DA)-100 mesh, hydrotalcite (HT), sodium nitrate, and sodium nitrite were purchased from Sigma Aldrich Chemical Company (Sigma Aldrich Chemical Co., Seoul, Korea). Other analytical-grade chemicals, such as sodium phosphate, potassium phosphate, sodium chloride, potassium chloride, sodium hydroxide, hydrochloric acid, sodium bicarbonate, magnesium sulfate, sodium sulfate, and calcium chloride, were used without further purification.

### 2.2. Measurements of Nitrate

#### 2.2.1. Use of Devarda’s Alloy

One hundred and fifty milligrams (150 mg) of Devarda’s alloy was used to convert 1 mL of nitrate solution (1–100 mM) to ammonia. Ten microliters (10 µL) of NaOH (12.5 M) was added to make an alkaline sample. The reaction time was 20 min, and transparent samples were collected for measurements.

#### 2.2.2. Preparation of the O17-EC Membrane

The O17-EC membrane was prepared following the approach used in our previous study [22] by mixing 15 µL of O17 stock (2 mg/mL) with 300 µL of 10 wt % EC in ethanol. The mixture was incubated for 4 h at room temperature and then coated on the bottom of the wells of a 96-well microtiter plate (NUNC Co. Copenhagen, Denmark). The O17-EC membrane was then dried at 60 °C for 24 h.

#### 2.2.3. Preparation of the O17-HT-EC Membrane

In this case, the clay HT was first mixed with O17 stock (2 mg/mL) and then 10 mg of the O17-adsorbed HT were mixed with 300 µL of 10 wt % EC in ethanol and the mixture was incubated for 2 h at room temperature. About 15 µL of the mixture was coated on the bottom of a well of a 96-well microtiter plate and then dried at 60 °C for 24 h. The O17-HT-EC membrane was used directly for nitrate measurement with the addition of 2 µL of NaOH (12.5 M) for each 200 µL of nitrate solution.

#### 2.2.4. Measurements

Nitrate concentrations were determined via the fluorescence intensity of the O17-EC membrane or the O17-HT-EC membrane at two emission wavelengths (*λ*_em_ = 555 nm and *λ*_em_ = 620 nm) with an excitation wavelength of 460 nm (*λ*_ex_ = 460 nm). The fluorescence spectra for detecting nitrate were measured using a multifunctional fluorescence microplate reader (Safire^2^, Tecan Austria GmbH, Wien, Austria). Reversibility of the O17-HT-EC membrane was performed with 1 mM nitrate and distilled water. The O17-HT-EC membrane was first exposed to distilled water and subsequently 1 mM nitrate solution. The microplate reader was set for fluorescence measurements against time with intervals of 30 s. The long-term stability of the O17-HT-EC membrane with various nitrate concentrations was evaluated through its repeatability by measuring the fluorescence intensity obtained initially and after 1 month. The effects of interferences on the O17-HT-EC membrane were investigated with nitrite (NO_2_^−^) at the same concentrations of nitrate. Artificial wastewater containing various nitrate concentrations was prepared, and the nitrate concentrations were determined with the O17-HT-EC membrane. The artificial wastewater included 2.5 mM CaCl_2_, 45 mM NaCl, 3.5 mM KH_2_PO_4_, 3.5 mM K_2_HPO_4_, 2.5 mM NaHCO_3_, 1 mM MgSO_4_, 2.5 mM Na_2_SO_4_, and nitrate in the concentration range of 0.1–1 mM.

### 2.3. Ratiometric Method

The ratiometric method was based on the ratio of the fluorescence intensities (FI) of the O17-EC membrane or the O17-HT-EC membrane at emission wavelengths (*λ*_em_ = 555 nm (FI_555_) and *λ_em_* = 620 nm (FI_620_)) as shown in Equation (1).

R = FI_555_/FI_620_ (1)

### 2.4. Colorimetric Method

Colorimetric measurements of the visual color transition of the O17-EC membrane as well as the O17-HT-EC membrane were performed under the irradiation of sunlight or a light-emitting diode at an excitation wavelength of 470 nm (LED470) of a microscope fluorescence camera (AM4115T-GRFBY Dino-Lite Edge: *λ*_ex_ = 470 nm and *λ*_em_ = 510 nm, AnMo Electronics Co., New Taipei, Taiwan) at the given nitrate concentrations.

### 2.5. Data Analysis

When adding interference to a given concentration of nitrate, the differences in the ratio of fluorescence intensities were assessed by one-way analysis of variance (ANOVA). Significant differences between samples were accepted with *p*-values of < 0.05. Statistical tests were performed using the InStat software (vers.3.01, GraphPad Software Inc, San Diego, CA, USA).

## 3. Results and Discussion

### 3.1. Choice of Materials

Ammonia could be produced through the reductive reaction of nitrate by the presence of Devarda’s alloy in alkaline solution as shown in Equation (2) [14,15],

3NO_3_^−^ + 8Al + 5OH^−^ + 18H_2_O → 3NH_3_ + 8[Al(OH)_4_]^−^ (2)

The produced ammonia in Equation (2) easily self-dissociates in aqueous solution to form ammonium cations that are usually determined via the colorimetric method or typical electrochemical methods. Quantitative analysis of ammonium cations in this study was based on our previous study for ammonia detection using the O17-EC membrane [22]. The O17-EC membrane has shown high sensitivity to ammonia in solution as well as long-term stability. In particular, analytical results are presented via a ratiometric fluorescence method, which is a low-cost, versatile, and useful method for continuous monitoring of ammonia in water. As is the case in the area of fluorescence-based sensors, measurements of fluorescence intensity at a single band edge are known to be problematic in practical applications [23]. The use of the ratiometric fluorescence method is a useful solution for correcting a variety of analyte-independent factors in fluorescent sensors. The temporal and spatial distribution of the measured fluorescence intensity in a fluorescent sensor can typically fluctuate due to an unequal distribution of fluorophores within the sensor, the variation in dynamics of fluorophores in different mediums, or noise in the measurement system, such as variations in the illumination intensity. Because of the self-calibration property of the ratiometric method, a wide range of ratiometric fluorescent sensors have been developed in order to allow for a precise and quantitative analysis [24,25,26,27]. Therefore, concentrations of nitrate could be determined by using the O17-EC membrane. The reduction of nitrate occurred due to the presence of aluminum (Al)-containing compounds. Devarda’s alloy (DA) contains aluminum (44–46%) and can act as a reducing reagent for the reductive reaction of nitrate. However, since the size of DA is not uniform and the color is black, the immobilization of DA into the O17-EC membrane could interfere with the fluorescence measurement and result in an unreliable method for ammonia detection. Herein, the reduction of nitrate with DA has been performed separately in a vial, and the O17-EC membrane was exposed to the transparent solution collected after nitrate reduction. In contrast to Devarda’s alloy, the clay HT is a fine white powder and consists of both positively charged brucite-like layers (Mg_6_Al_2_(OH)_16_^2+^), which could participate in the reduction of nitrate, and negatively charged inter-layers (CO_3_·4H_2_O^2−^) that maintain the alkaline medium. Therefore, the clay HT was immobilized into the O17-EC membrane and showed insignificant effects on the fluorescence measurement.

### 3.2. Response of the O17-EC Membrane after Nitrate Reduction with DA

**Figure 1:**
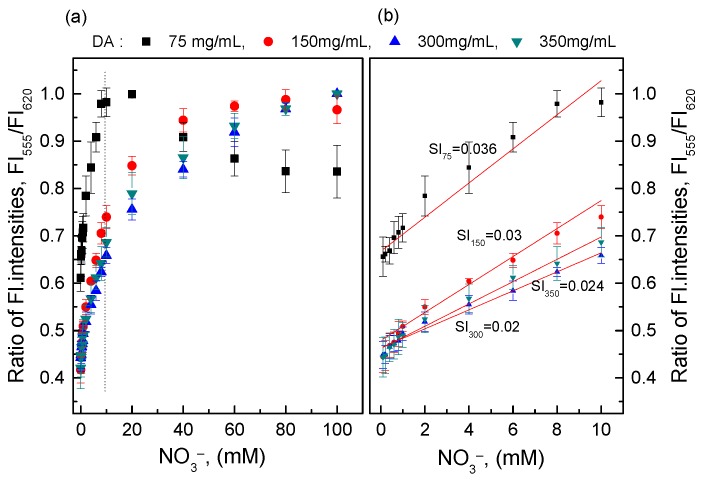
(**a**) The response of the oxazine170 perchlorate–ethyl cellulose (O17-EC) membrane to the ammonia produced from the reductive reaction of different nitrate concentrations with different amounts of Devarda’s alloy (DA); (**b**) Slope (SI) from linear calibration curves of the O17-EC membrane in the nitrate concentration range of 0.1–10 mM.

Different amounts of DA were first employed to study the efficiency of DA on the reductive reaction of nitrate in a wide range of nitrate concentrations (0.1–100 mM). As shown in Figure 1 (inset), the slope value (SI) indicates the sensitivity of the O17-EC membrane to nitrate concentrations. The slope value (SI) with 75 mg DA was highest at the nitrate concentration range of 0.1–10 mM, but 75 mg DA was not sufficient for the reductive reaction of nitrate at a higher nitrate concentration of 10 mM (Figure 1). Therefore, 150 mg DA was chosen for nitrate reduction, since the SI with 150 mg DA was the best one among the high amounts of the used DA and its calibration curve was continuously developed as an exponential function at nitrate concentrations higher than 10 mM.

**Figure 2:**
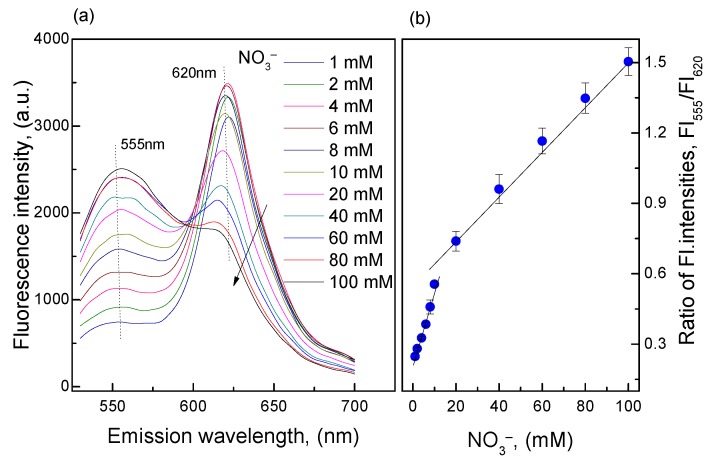
(**a**) Fluorescence emission spectra of the O17-EC membrane when exposed to ammonia produced from the reductive reaction of different nitrate concentrations in the range of 1–100 mM; (**b**) Calibration curve of the O17-EC membrane in the nitrate concentration range of 1–100 mM.

Figure 2 shows that large amounts of ammonia were produced from the reductive reaction of nitrate in the concentration range of 1–100 mM and the O17-EC membrane responded well to these amounts of ammonia. The calibration curve collected from the ratio of fluorescence intensities at peaks of *λ*_em_ = 555 and 620 nm (FI_555_/FI_620_) shows two linear detection ranges for nitrate: 1–10 mM (*r*^2^ = 0.989) with an LOD = 0.346 mM and 10–100 mM (*r*^2^ = 0.981) with an LOD = 9.89 mM. According to our previous study [22], the O17-EC membrane can detect ammonia in the range of 0.06–3.5 mM, but in this case when the nitrate concentration was lower than 1 mM, the sensitivity of the O17-EC membrane was very low compared with the sensitivity of the O17-EC membrane at nitrate concentrations greater than 1 mM. This is due to the loss of ammonia produced from the reductive reaction of nitrate to the ammonia measurement, and subsequently the remaining ammonia concentration reached the minimal threshold of this range. However, the wide detection ranges of the O17-EC membrane to nitrate shown in Figure 2 also indicated that the O17-EC membrane can extend the linear detection range for ammonia in some typical conditions, since the moles of nitrate and ammonia are equal amounts in Equation (2).

### 3.3. Response of the O17-HT-EC Membrane to Nitrate

#### 3.3.1. Optimal Amount of HT

**Figure 3:**
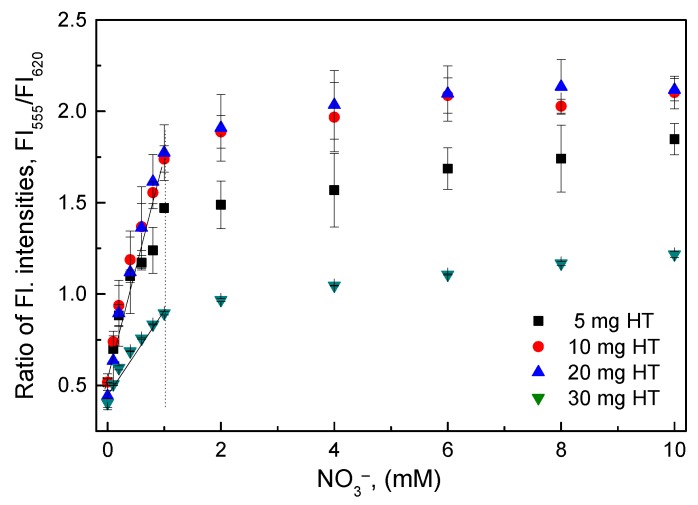
Response of the O17-HT-EC membranes immobilized with different amounts of hydrotalcite (HT) to various nitrate concentrations.

The O17-HT-EC membranes that were immobilized with different amounts of HT (5, 10, 20, and 30 mg) were sensitive to nitrate in the concentration range of 0.1–10 mM (Figure 3). Five milligrams (5 mg) HT was sufficient for the reductive reaction of nitrate at high concentrations, but the use of 30 mg HT was redundant, leading to a decrease in sensitivity of the O17-HT-EC membrane. The O17-HT-EC membrane immobilized with 20 mg HT had the highest sensitivity in the nitrate concentration range of 0.1–1 mM among the four amounts of HT. However, the O17-HT-EC membrane immobilized with 10 mg HT could be considered to have the optimal amount of HT, because it had almost the same sensitivity as the membrane with 20 mg HT.

#### 3.3.2. Calibration Curve of the O17-HT-EC Membrane

**Figure 4:**
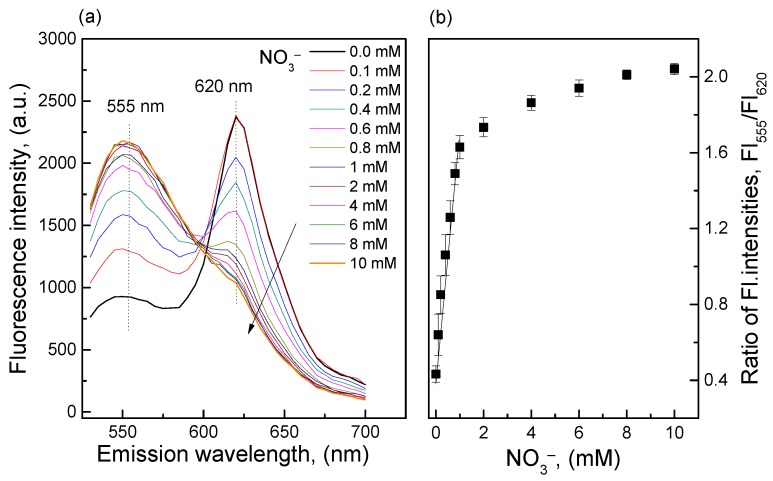
(**a**) Fluorescence emission spectra of the O17-HT-EC membrane when exposed to different nitrate concentrations; (**b**) Calibration curve of the O17-HT-EC membrane in the nitrate concentration range of 0.1–10 mM.

Because the clay HT was combined with O17 and EC in a thin membrane, the ammonia produced from the reductive reaction of nitrate was immediately measured by an ammonia-sensitive component (dye O17) of the O17-HT-EC membrane. Therefore, the loss of ammonia from the O17-HT-EC membrane could be prevented, and the membrane could recognize ammonia produced from low concentrations of nitrate as shown in Figure 4. The linear detection range was 0.1–1 mM with an LOD of 0.089 mM. The direct immobilization of HT in the matrix of the O17-EC membrane created a new version of the O17-EC membrane that showed the possibility for nitrate analysis in a suitable detection range of limited nitrate concentrations in drinking water according to the standard set by the EPA of the United States [1]. Although the O17-HT-EC membrane was advantageous during measurements of low nitrate concentrations, it also showed a few limitations, such as a longer time for returning back to the background signal compared with the use of the O17-EC membrane only. This is due to the slow release of NH_4_^+^OH^−^ in the CO_3_·4H_2_O^2−^ layers of the clay HT when cleaning the O17-HT-EC membrane.

#### 3.3.3. Reversibility of the O17-HT-EC Membrane

**Figure 5:**
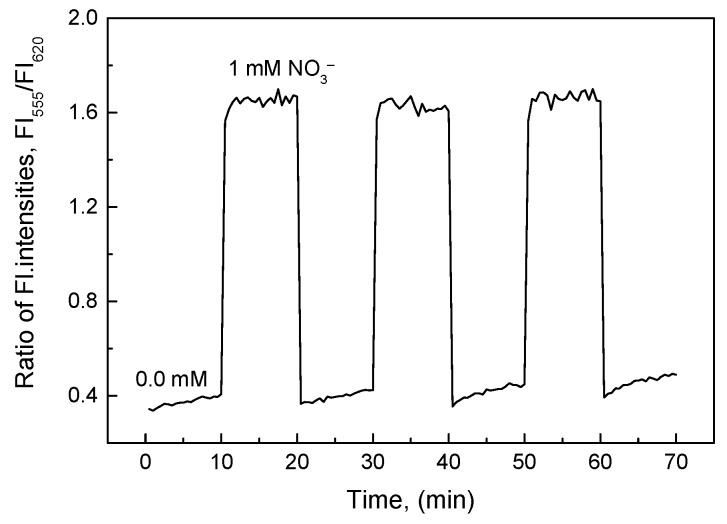
Reversibility of the O17-HT-EC membrane when repeatedly exposed to a sequence of distilled water (0.0 mM) and 1 mM nitrate.

Reversibility of the O17-HT-EC membrane was quite precise, as the relative standard deviation (RSD) was very small, i.e., 3.5% in distilled water (0.0 mM) and 1.9% at 1.0 mM nitrate (Figure 5). However, the time taken to clean the O17-HT-EC membrane after each measurement was longer than that for the O17-EC membrane only. This is attributed to the alkaline preference of the clay HT that slowly releases ammonium cations from the pair of NH_4_^+^O17^−^. The reaction between O17 and ammonium cations was clearly described in our previous study [22]. While the response time of the O17-HT-EC membrane was very short (about t_95_ = 10 s) at all nitrate concentrations measured in this work, about 6 min (t_95_ = 6 min) was required to achieve 95% of the maximal value in distilled water (0.0 mM).

#### 3.3.4. Interference of Nitrite with the O17-HT-EC Membrane

**Figure 6:**
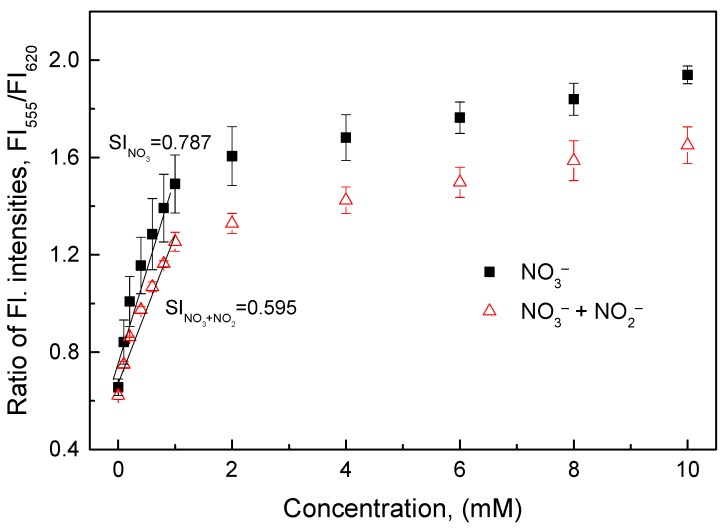
Response of the O17-HT-EC membrane when exposed to nitrate solutions containing nitrite with the same nitrate concentrations.

The sensitivity of the O17-HT-EC membrane decreased significantly when both nitrate and nitrite ions (NO_2_^−^) with the same concentrations were present in the aqueous solutions (Figure 6). The sensitivity of the O17-HT-EC membrane in the concentration range of 0.1–1 mM (SI = 0.595) decreased by about 24% as compared with its sensitivity to nitrate only (SI = 0.784). This response of the O17-HT-EC membrane occurred continuously at higher concentrations of nitrate and nitrite over 1 mM.

#### 3.3.5. Long-Term Stability of the O17-HT-EC Membrane

**Figure 7:**
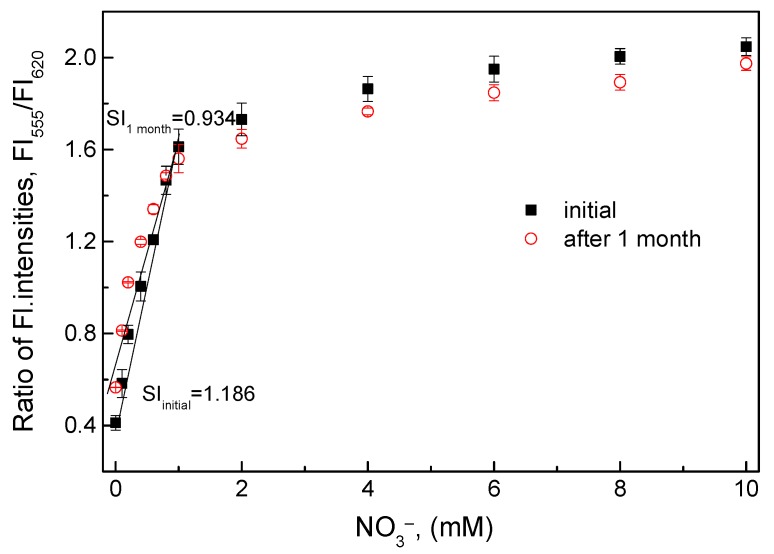
Change in the ratiometric fluorescence intensities of the O17-HT-EC membrane in the nitrate concentration range of 0.1–10 mM at initial use and after 1 month.

The O17-HT-EC membrane was undamaged after 1 month of continuous use and showed high sensitivity to nitrate (Figure 7). However, the slope value (SI = 0.934) of the calibration curve decreased by about 21.2% as compared with the slope value (SI = 1.186) at the initial use in the nitrate concentration range of 0.1–1 mM. As mentioned above, the clay HT could release ammonium cations slowly when being cleaned at each measurement. Also, during the measurements of nitrate for the evaluation of other parameters, a small amount of ammonium cations was conjugated with negatively charged layers (CO_3_·4H_2_O^2−^) of the clay HT in the O17-HT-EC membrane. This led to an increase in the fluorescence intensity at an emission wavelength of 555 nm, resulting in an increase of the ratiometric fluorescence intensity at the background signal as shown in Figure 7.

### 3.4. Measurement of Artificial Waste Water

**Figure 8:**
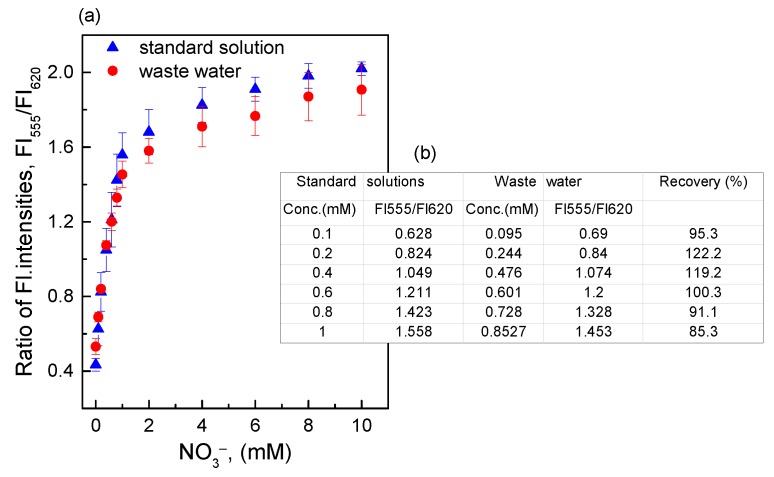
(**a**) Response of the O17-HT-EC membrane to different nitrate concentrations in standard solutions and artificial waste water; (**b**) Table for the recovery of the O17-HT-EC membrane when exposed to different nitrate concentrations in artificial waste water.

From the response of the O17-HT-EC membrane when exposed to nitrate in the standard solutions (Figure 8) we obtained the linear equations, such as Y (ratiometric fluorescence intensity) = 1.008 × X (nitrate concentration) + 0.594 (*r*^2^ = 0.982), for the O17-HT-EC membrane. Based on the linear equations, the concentrations of nitrate present in artificial waste water were calculated as shown in Figure 8 (inset table). The recovery of the O17-HT-EC membrane obtained was about 85–122% in the nitrate concentration range of 0.1–1.0 mM. This was achieved due to the presence of salts in artificial waste water, which prevented the reduction of nitrate to ammonia. According to Daniel et al. [5], the salinity of water has significant effects on the reaction kinetics of nitrate. That is, low salinity (>10 µM) leads to faster reaction development, but the reduction of nitrate is inhibited at high salinity. Thus, the NaCl concentration of 4.5 mM in this work could be one of the reasons for the interference in the reduction of nitrate, leading to a low response of the O17-HT-EC membrane to the smaller amount of ammonia produced.

Any method of detecting the end product of nitrate reduction should account for the presence of nitrite or ammonia in unknown samples, particularly in wastewater samples. The ammonia concentration of the unknown sample is measured first and then the ammonia concentration is subtracted from the concentration of the nitrate concentration.

Table 1 shows some typical issues associated with nitrate detection, in which the analytical parameters are compared with those of the ratiometric fluorescence nitrate sensors developed in this study. In this comparison, the ratiometric fluorescence nitrate sensors exhibited good analytical performance, specifically in terms of high sensitivity and selectivity, a fast response time, and long-term stability.

### 3.5. Colorimetric Measurements of the Nitrate-Sensing Membranes

**Figure 9:**
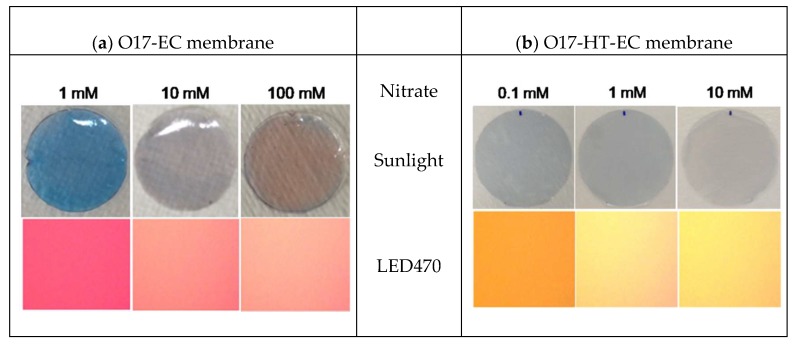
The visual color transitions of (**a**) the O17-EC membrane and of (**b**) the O17-HT-EC membrane when being exposed to different nitrate concentrations under sunlight (round images) or under light-emitting diode at an excitation wavelength of 470 nm (LED470) irradiation (square images).

The visual color transition of the O17-EC membrane under sunlight or LED470 irradiation of a microscope fluorescent camera at different nitrate concentrations is clearly shown in Figure 9a. Under sunlight, the original blue color of the O17-EC membrane was kept at 1 mM nitrate and then changed toward a purple color when increasing the nitrate concentration to 10 mM with an especially clear exhibition at 100 mM (round images). The color transition of the O17-EC membrane via fluorescence emission of the dye O17 under LED470 irradiation was also observed since the red color of O17 at *λ*_em_ = 620 nm decreased to a pink color when increasing the nitrate concentrations to 10 mM and 100 mM (square images). The shift in the emission wavelength of the O17-EC membrane when reacting with ammonia produced from the reduction of nitrate is the reason for such changes in color. Herein, the reaction mechanism of the color transition of O17 is explained by the proton donation of the weakly acidic oxazine to the proton acceptance of the weak base, in which the addition of ammonium hydroxide solution to oxazine perchlorate dye produces a change in color from blue to purple due to the deprotonation of one of the amino groups. The sequence of reactions of ammonia with oxazine dye is predicted in Equations (3)–(5):

NH_3_ + H_2_O ↔ NH_4_^+^OH^−^ (3)

NH_4_^+^OH^−^ + H^+^Dye^−^ ↔ NH_4_^+^Dye^−^ + H_2_O (4)

NH_4_^+^Dye^−^ ↔ H^+^Dye^−^ + NH_3_ (5)

In the reaction scheme, the hydroxyl radical abstracts a loosely bound proton when the positively charged ammonium cation leaves to counterbalance the perchlorate anion. This reaction produces significant fluorescence band shifts, which can be accounted for by a semi-classical oscillator model predicting two dichromic electronic band systems associated with this dye [22]. In distilled water or at a low ammonia concentration (e.g., 1 mM), the blue color of the O17-EC membrane still remained visible, but at higher ammonia concentrations the blue color changed to a purple color, a process shown by Equation (4).

The visual color transition of the O17-HT-EC membrane is shown in Figure 9b. Under sunlight, the color transition of the O17-HT-EC membrane was unclear between 0.1 mM and 1 mM nitrate, but the light blue color of the membrane tended toward a light purple color at 10 mM nitrate (round images). Different from the color transition under sunlight, the color transition of the O17-HT-EC membrane under LED470 irradiation was clearly observed (square images). The orange color of the O17-HT-EC membrane at 0.1 mM nitrate was changed to a mixed color of light yellow and pink at 1 mM nitrate and changed to a yellow color at 10 mM nitrate.

## 4. Conclusions

The O17-EC membrane was successfully applied for colorimetric and fluorescent analysis of nitrate in aqueous solution in a wide detection range of 0.1–100 mM through the reductive reaction of nitrate in the presence of either Devarda’s alloy or the clay hydrotalcite. The method of using aluminum-containing compounds created different sensing membranes with different detection ranges of nitrate. These include the O17-HT-EC membrane with a low detection range of 0.1–1.0 mM and a detection limit of 0.089 mM and the O17-EC membrane with a high detection ranges of 1–10 mM and 10–100 mM. The nitrate-sensing membranes also exhibited high sensitivity, a fast response time (~10 s), long-term stability (>1 month), excellent reversibility, and clear color transition of the sensing membranes under sunlight as well as under LED470 irradiation, such that it emerges over other fluorescent sensors. The simple and easy fabrication of the nitrate-sensing membranes by combining the O17-EC membrane with aluminum-containing compounds is intended to replace the complicated methods currently in use, such as flow injection analysis (FIA) or molecular emission cavity analysis (MECA) for nitrate analysis in certain cases. The precise analysis of the O17-EC membrane or the O17-HT-EC membrane for nitrate in wastewater is valid for practical applications, such as nitrate detection in environmental samples.

## Figures and Tables

**Table 1:** Summary of certain nitrate sensors.

Detection Method	Direct Detection of NO_3_^−^	Detection Range	Detection Limit	Long-Term Stability	Ref.
Griess	No (via NO_2_)	0–100 µM	0.45 µM	N/A	[5]
Electrode	Yes	0.01–1 mM	N/A	N/A	[10]
Enzyme-Griess	No (via NO_2_)^−^	3.57–35 7 µM	0.43 µM	N/A	[12]
Devarda’s alloy-MECA	No (via NH_3_)	0.7–28.5 mM	71 µM	N/A	[15]
Zinc-BAWIS	No (via NH_3_)	0.002–1 mM	1.7 µM	N/A	[16]
Ion selective electrode (ISE)	Yes	10–100 µM	1.1 µM	N/A	[18]
Fluorescent probe	Yes	0–100 µM	0.475 µM	N/A	[20]
Disposable fluorescent film	Yes	0.1–100 mM	0.1–1 mM		[21]
DA and HT fluorescent membranes	No (via NH_3_)	1–10 mM and 10–100 mM 0.1–1 mM	0.346 mM and 9.89 mM 0.089 mM	>1 month	This study

BAWIS, bulk acoustic wave impedance sensor; MECA, molecular emission cavity analysis; N/A, Not Available.
